# Use of a *Plasmodium vivax* genetic barcode for genomic surveillance and parasite tracking in Sri Lanka

**DOI:** 10.1186/s12936-020-03386-3

**Published:** 2020-09-21

**Authors:** Rajika L. Dewasurendra, Mary Lynn Baniecki, Stephen Schaffner, Yamuna Siriwardena, Jade Moon, R. Doshi, Sharmini Gunawardena, Rachel F. Daniels, Daniel Neafsey, Sarah Volkman, Naduviladath V. Chandrasekharan, Dyann F. Wirth, Nadira D. Karunaweera

**Affiliations:** 1 Department of Parasitology, Faculty of Medicine, University of Colombo, 25, Kynsey Road, Colombo 8, Sri Lanka; 2 Infectious Disease and Microbiome Program, Broad Institute of MIT and Harvard, Cambridge, MA 02142 USA; 3 Department of Immunology and Infectious Diseases, Harvard T. H. Chan School of Public Health, Boston, MA 02115 USA; 4 Department of Organismic and Evolutionary Biology, Harvard University, Cambridge, Boston, MA 02138 USA; 5 Department of Public Health, John Hopkins University, Baltimore, MD 21218 USA; 6 Department of Chemistry, Faculty of Science, University of Colombo, Munidasa Kumaratunga MW, Colombo 3, Sri Lanka

**Keywords:** High-resolution melting method, Genetic diversity, SNP barcode

## Abstract

**Background:**

Sri Lanka was certified as a malaria-free nation in 2016; however, imported malaria cases continue to be reported. Evidence-based information on the genetic structure/diversity of the parasite populations is useful to understand the population history, assess the trends in transmission patterns, as well as to predict threatening phenotypes that may be introduced and spread in parasite populations disrupting elimination programmes. This study used a previously developed *Plasmodium vivax* single nucleotide polymorphism (SNP) barcode to evaluate the population dynamics of *P. vivax* parasite isolates from Sri Lanka and to assess the ability of the SNP barcode for tracking the parasites to its origin.

**Methods:**

A total of 51 *P. vivax* samples collected during 2005–2011, mainly from three provinces of the country, were genotyped for 40 previously identified *P. vivax* SNPs using a high-resolution melting (HRM), single-nucleotide barcode method. Minor allele frequencies, linkage disequilibrium, pair-wise F_ST_ values, and complexity of infection (COI) were evaluated to determine the genetic diversity. Structure analysis was carried out using STRUCTURE software (Version 2.3.4) and SNP barcode was used to identify the genetic diversity of the local parasite populations collected from different years. Principal component analysis (PCA) was used to determine the clustering according to global geographic regions.

**Results:**

The proportion of multi-clone infections was significantly higher in isolates collected during an infection outbreak in year 2007. The minor allele frequencies of the SNPs changed dramatically from year to year. Significant linkage was observed in sample sub-sets from years 2005 and 2007. The majority of the isolates from 2007 consisted of at least two genetically distinct parasite strains. The overall percentage of multi-clone infections for the entire parasite sample was 39.21%. Analysis using STRUCTURE software (Version 2.3.4) revealed the high genetic diversity of the sample sub-set from year 2007. In-silico analysis of these data with those available from other global geographical regions using PCA showed distinct clustering of parasite isolates according to geography, demonstrating the usefulness of the barcode in determining an isolate to be indigenous.

**Conclusions:**

*Plasmodium vivax* parasite isolates collected during a disease outbreak in year 2007 were more genetically diverse compared to those collected from other years. In-silico analysis using the 40 SNP barcode is a useful tool to track the origin of an isolate of uncertain origin, especially to differentiate indigenous from imported cases. However, an extended barcode with more SNPs may be needed to distinguish highly clonal populations within the country.

## Background

**Fig. 1:**
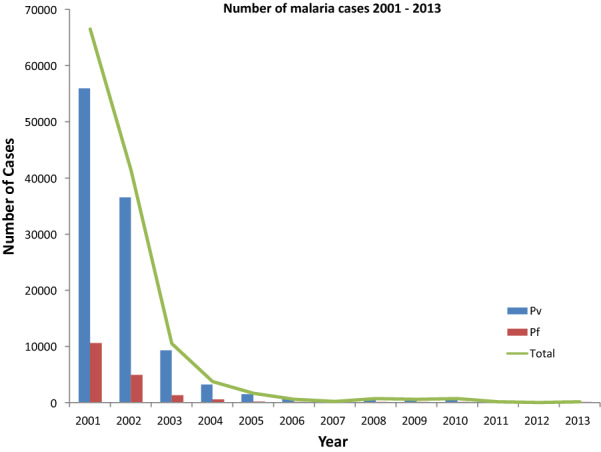
Number of malaria cases reported 2001–2013. (Source: Anti-malaria Campaign, Ministry of Health, Sri Lanka)

Malaria imposed a tremendous health burden in Sri Lanka, until the year 2000 [1, 2] (Fig. 1). The key factors that contributed to the successful reduction of malaria incidence included successful integrated vector control, entomological and parasitological surveillance, thorough epidemiological investigations, disease management, and financial support for diagnostic laboratories and health education [3]. Suppression of control efforts, upon approaching near elimination, often leads to malaria resurgence [4, 5], which is clearly evident from past experiences of many countries that include Sri Lanka [1, 2], India [6] and Madagascar [7].

In 2016, the World Health Organization (WHO) certified Sri Lanka as malaria-free. However, as the past predicts, there remains a risk of malaria re-introduction. The last indigenous case of malaria in Sri Lanka was reported in October 2012; however, imported malaria cases continue to be recorded in notable numbers (95, 49, 36, 41, 47, and 48 cases reported during successive years between 2013 and 2018) [5, 8]. Thus, it is crucial to maintain active surveillance programmes to detect such cases early and contain the situation effectively. Accurate identification of the parasite is critically important in this process in order to make proper patient management decisions especially regarding chemotherapy [9].

High-resolution melting (HRM) analysis has been used to develop single nucleotide polymorphism (SNP) barcodes for both *Plasmodium falciparum* and *Plasmodium vivax* parasites [10, 11]. HRM is a simple and cost-effective method for genotyping and mutation scanning [12, 13]. The malaria molecular barcodes have been shown to have the sensitivity to identify an infection with a single parasite genome or complex (multi-clone infections) parasite infections containing mixtures of parasite genomes within a given sample [9]. Furthermore, genetic barcodes have been used and validated as tools to monitor transmission and to evaluate interventions showing its capacity to make an impact on malaria elimination efforts [14]. The study of the genetic structure/composition of the parasite will be useful in understanding the history of the parasite populations that may enable tracking down the parasites to its origin, as well as providing insights into trends in parasite transmission which are vital and valuable information for elimination programmes [15].

The main objectives of the study described were to investigate the genetic diversity of *P. vivax* parasite isolates collected from patients mainly from three different locations in Sri Lanka over a period of 7 years (2005–2011), and to test the utility of a SNP barcode in tracking parasites to its origin, which remains particularly important in post-elimination settings, such as in Sri Lanka.

## Methods

### Ethical considerations

Ethics approval for this study was obtained by the Ethics Review Committee, Faculty of Medicine, University of Colombo (Ref No: EC–11–191).

### Field isolates

Before elimination phase, the Anti-malaria Campaign, Ministry of Health, Sri Lanka collected blood samples of all malaria-positive patients through the Regional Malaria Officers from all of the 25 administrative districts in Sri Lanka. According to a mutual agreement between the Anti-malaria campaign and the Department of Parasitology, Faculty of Medicine, University of Colombo, a dried blood spot on filter paper (3MM Whatmann) from these blood samples collected were sent to the laboratory of the Department of Parasitology for research purposes (after obtaining informed consent from the patient), together with the records of the age, gender and location of the patient. These patients presented with fever and were diagnosed as having vivax malaria infection confirmed through microscopy in local health facilities. All samples were assumed to be autochthonous at the outset, since a travel history outside the country was not mentioned for any patient.

**Fig. 2:**
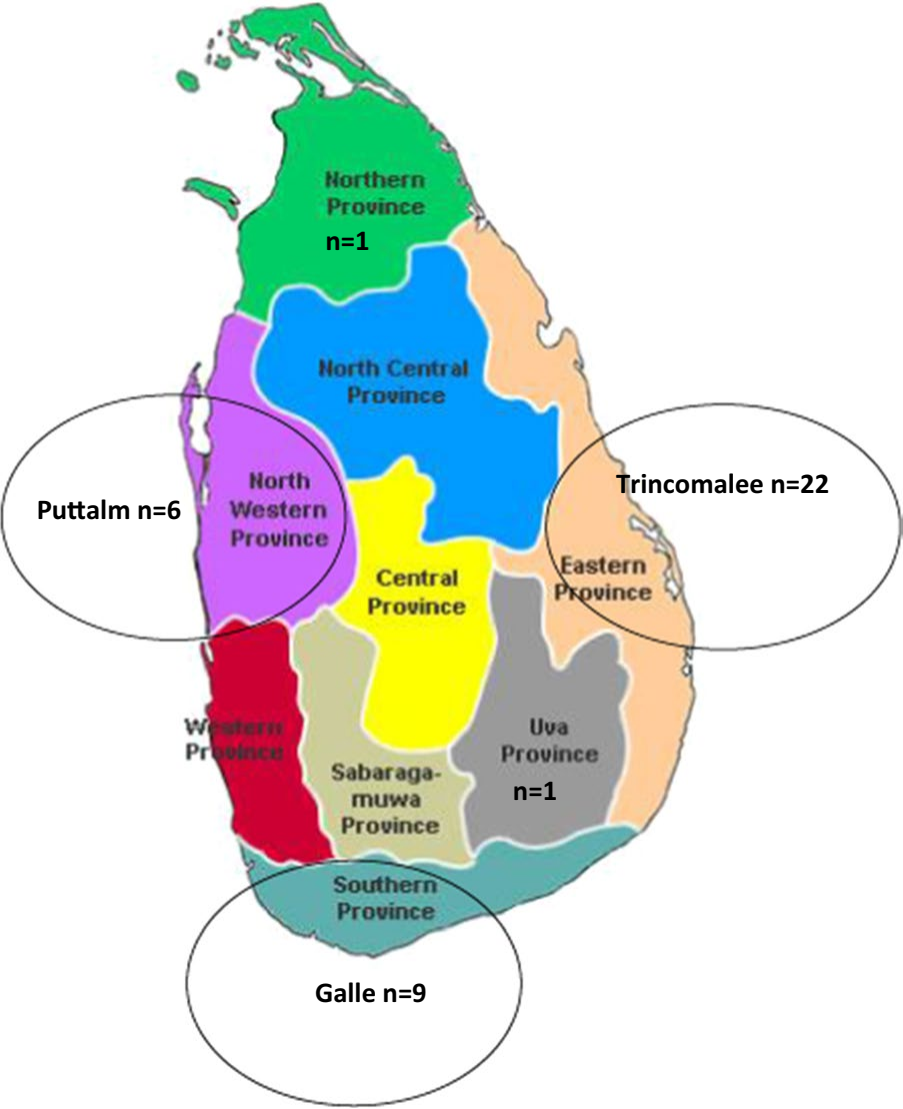
The locations/districts from where the parasite isolates were collected. The isolates were collected from three districts in Sri Lanka; Trincomalee (Eastern province), Puttalm (North Western province), and Galle (Southern province). The locations of 12 parasite isolates were not recorded

Fifty-one *P. vivax* field isolates which were sent to the Department of Parasitology, by the Anti-malaria campaign were selected for genotyping (Additional file 1). These isolates had been collected between 2005 and 2011 from the Eastern (n = 22), North Western (n = 6), Southern (n = 9), Uva (n = 1), and Northern (n = 1) provinces. The origin or location of 12 parasite field isolates was not recorded (Fig. 2).

### DNA sample preparation and HRM barcode method

DNA was extracted from the dried blood spots using a commercial kit (QIAmpBlood Micro Kit—cat# 56304, QIAGEN, Germany) according to the manufacturer’s protocol. Extracted DNA samples (each 1 μL) were subjected to whole genome amplification (WGA) using the IllustraGenomiPhi V2 DNA Amplification kit (GE Healthcare Bio-Sciences, Piscataway, NJ, USA) according to the manufacturer’s protocol, yielding 40 μL of amplified material. The amplified DNA was then purified using the AgencourtAMPure SP system (Beckman Coulter, Inc., Beverly, MA, USA) according to the manufacturer’s protocol and the samples were quantified using a NanoDrop3300 Fluorospectrometer (Thermo Scientific, Waltham, MA, USA). The samples were then diluted in 1× Tris–EDTA (TE) Buffer (VWR, Radnor, PA, USA) to a concentration of 1 ng/μL.

The *P. vivax* HRM barcode was developed as described by Baniecki et al*.* [11]. The present study used 40 of the 42 SNPs (Additional file 2). In brief, an HRM master mix was prepared with 1.0 μL of forward primer (1.0–0.5 μM/μL), 1.0 μL of reverse primer (1.0–0.5 μM/μL), 1.0 μL of PCR-grade water and 4.0 μL of 2.5× Light Scanner Master mix (BioFire Diagnostics Inc. Salt Lake City, Utah, USA). The optimal primer pair concentration of the forward and reverse primers depended on the individual HRM assays [11]. To each well of a 384-well PCR plate, 7.0 μL of the master mix and 3.0 μL of template DNA (1 ng/μL) after WGA (details described in Baniecki et al*.* [11]) were added in a total volume of 10.0 μL, The plate was sealed with an optically clear, pressure-sensitive adhesive film and was centrifuged at 1000 rpm for 1 min. HRM was performed on a QuantStudio6 Flex Real-Time PCR system (Applied Biosciences). The two-step PCR cycling conditions were as follows:

95 °C for 120 s, 40 cycles of 94 °C for 30 s and 64 °C for 60 s followed by a final HRM cycle of 95 °C, 55 °C and 95 °C each of 15 s.

### Genotype determination

Method described in Baniecki et al. was used to determine the genotypes [11]. Briefly, a control sample was included for each test sample in the HRM assay to identify the reference and alternate SNP temperature melt-curves (T_m_) of each SNP in the test samples. The T_m_ peak produced by the control sample was aligned with the peaks produced by the test samples to identify each allele. Sample genotypes were identified using the derivative T_m_ curve for each SNP of each sample. The reference/alternate alleles produce a single T_m_ peak that differs 0.7–1.2 °C and a monomorphic SNP can be identified by aligning this single T_m_ peak with the control T_m_ curve. Since *P. vivax* parasites are haploid in peripheral blood stream identification only a single allele in any SNP position is considered as a monomorphic site, while detection of 2 alleles is considered as a polymorphic site. Polymorphic sites produces skewed or shifted T_m_ curves. Samples with more than one polymorphic site in a given SNP position was considered multiclonal/polygenomic [15]. In polymorphic infections, when the isolate consisted of 2 clones, the allele for a given SNP position was determined by comparing the melting curve of the isolate with the relevant reference and alternate controls. However, the exact number of clones could not be determined using this method, which enables categorization of an isolate as either single clonal or multi-clone (or polyclonal).

### Data analysis

**Fig. 3:**
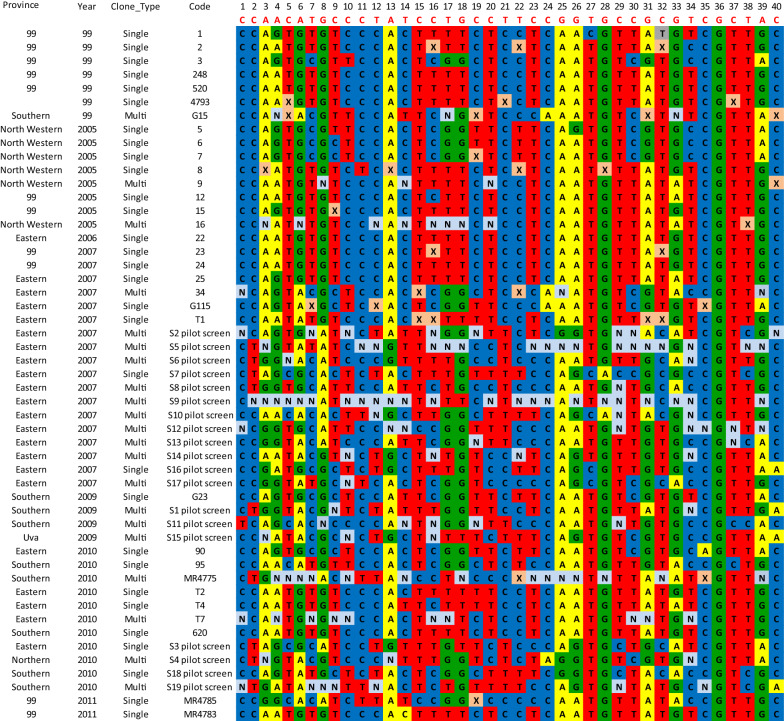
Barcodes of parasite isolates grouped together according to year of collection. The location and the year of collection (99 = unknown) for each isolate are indicated in the first two columns. Genotypes are indicated in colour codes: A = yellow, C = blue, G = green, T = red; N (light blue) denotes polygenomic sites, and X (light orange) denotes HRM assays that were failed

A total of 51 samples were genotyped by HRM. The samples were divided into 6 sub-sets according to the year of collection, as follows: sub-set of isolates collected in 2005 (n = 8), 2006 sub-set (n = 1), 2007 sub-set (n = 18), 2009 sub-set (n = 4), 2010 sub-set (n = 11), and 2011 sub-set (n = 2). The year of collection of seven samples were unknown. A genetic barcode for the Sri Lankan vivax malaria parasite was built by arranging SNP data for the 40 SNPs, grouped by each sample sub-set for each year (Fig. 3) and by arranging the SNPs grouped by the location from which the parasite isolates were collected (Additional file 3).

The haplotypes were developed by using Arlequin V 3.1 software by using ELB (Bayesian) algorithm [16] and further confirmed by the COIL web-based tool [17] which uses a Bayesian model to assign probabilities for SNP barcode data. This program can consider the probability of having a mixture of reference and alternate alleles (multi-clone infections). Furthermore, this program also allows usage of genotypic data with missing information for fewer than 3 loci. The program excludes the samples/isolates with more than 3 loci with missing information. Thus, three samples (one each from the sample sub-sets from 2005 and 2007, and another with unknown year of collection) where excluded from the COIL analysis. Haplotypes were used in generating the F_ST_ values to measure the pair-wise distances for determining genetic distance between sample sub-sets.

The minor allele frequency (MAF) was calculated for each polymorphic genotype by counting the reference and alternate alleles, with its average (the unweighted mean) calculated as described by Baniecki et al. [11]. The isolates from multi-clone infections were not considered in calculating the MAF for a particular SNP. These figures for MAF were confirmed by re-calculating the MAF by using the COIL web tool, while retaining the multi-clone isolates designated with an “N call”. Although both alleles of multi-clone isolates could be determined by using the differences in the melting curves, the frequency of either major/minor allele could not be determined using the HRM method. Therefore, the positions indicating having 2 or more alleles were designated with an “N call”. These data were retained in analysis by using COIL web-based tool predicting the minimum number of samples present [17]. Loci in linkage disequilibrium (LD) in each group, defined as non-random associations of alleles at each SNP, were calculated for polymorphic SNPs across all 40 SNPs at a significant level of p < 0.05 with Fisher’s exact test using Markov chain (chain length = 10,000, de-memorization = 10,000) [18–20]. Principal component analysis (PCA) was carried out using the online tool ClustVis [21] to compare the genetic structure of parasite isolates from different locations at different time points as well as to compare the genetic structure of parasite isolates collected from Sri Lanka (South Asian, n = 51) and other geographic regions, i.e., Thailand, Cambodia, Indonesia (Southeast Asian, n = 181) and Peru (South American, n = 69). The genotypic data were extracted from open access data from MalariaGEN *P. vivax* genome variation data release 2016 [22] and Cowell et al. 2018 data [23].

All 51 samples were considered for analysis; however, 7 of the 51 samples with unknown years of collection were excluded from linkage analysis and F_ST_ estimates. Since there was only one sample from 2006 and two from 2011, they were only included in establishing the barcode since the number of isolates is not a limiting factor for building the barcode. However, they were excluded from other analyses. All 51 samples were used in PCA together with the samples from other global geographic regions.

The genetic distance between the haplotypes was examined by calculating pair-wise genetic distance (F_ST_). The F_ST_ values were obtained using Arlequin V 3.1 software [16] where the genetic distance of pairs of populations were determined using Slatskin’s linearized method with 100 permutations with 100,000 steps in Markov chain and 10,000 de-memorization steps [24]. These values were further confirmed by generating F_ST_ values using the COIL web-based tool [17]. The significance level was 0.05. The proportion of multi-clone infections which reflects complexity of infection (COI) was determined to identify the number of genetically distinct parasite strains simultaneously infecting a patient using the COIL program as described previously [17].

A model-based Bayesian method was used to partition of individual parasite isolates into genetic clusters using the STRUCTURE V2.3.4 software [25]. The isolates were grouped into four groups based on the year they were collected (i.e., 2005, 2007, 2009/2010, 2011). The number of clusters (K) was determined by simulating K values from 1 (with no genetic differentiation among the groups) to 4 (genetic differentiation of each group). The posterior probability values of each K were estimated from a Markov Chain Monte Carlo (MCMC) method. The multi-clone isolates were excluded in structure analysis.

Additional data analysis was carried out using Excel for Windows 2013, SPSS v 19.0, Genepop v 4.6, and Tassel v 5.2.40 [26–28].

## Results

### Genetic barcodes of Sri Lankan *Plasmodium vivax* parasites

The *P. vivax* SNP barcode was used to screen all 51 samples in duplicate. The barcodes were arranged according to the year in which the corresponding isolate was collected (SNP date). The analysis revealed that the isolates collected during the earlier years, i.e., 2005, were less genetically diverse, with more or less constant/shared sub-sets of the haplotypes. In contrast, the sample sub-set from 2007 looked more diverse and with more multi-clone infections (Fig. 3). Secondly, the haplotypes were arranged according to the location from where the corresponding isolates were collected (Additional file 3). It was obvious that the isolates from the Eastern province and the Southern province exhibited a higher genetic diversity with more multi-clone infections compared to the isolates from the North-Western province. Interestingly, the isolates from the Eastern province and the Southern province were collected from short epidemics in 2007 from Trincomalee and 2009/10 from Galle districts, respectively.

**Fig. 4:**
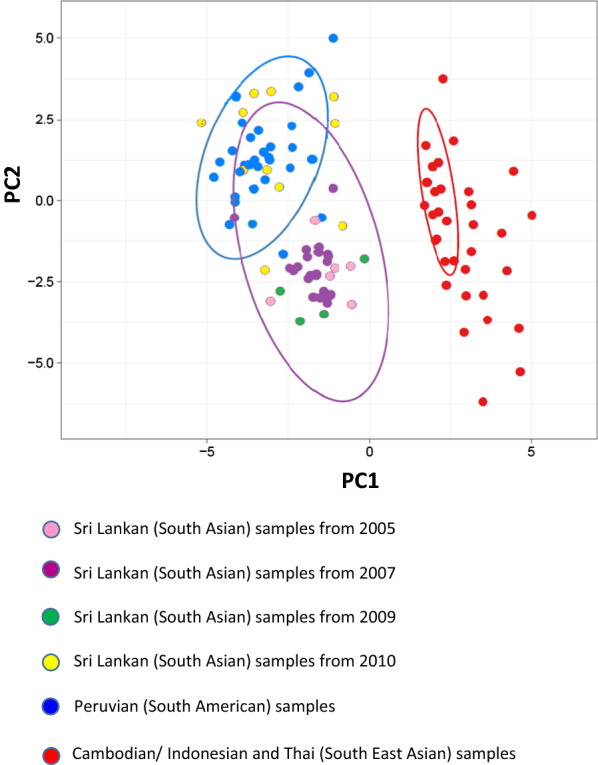
Principal component analysis (PCA) of isolates collected from different global geographic regions. South Asian (Sri Lanka), South American (Peru-Amazon) and Southeast Asian (Thailand, Cambodia, Indonesia)

This genetic barcode was used to distinguish the parasite populations from different geographic regions, i.e., Thailand. Cambodia, Indonesia (Southeast Asian) and Peru (South American) and Sri Lanka (South Asian) using PCA. The isolates from Cambodia, Thailand and Indonesia formed a single cluster, while the Peruvian (South American) and Sri Lankan (South Asian) isolates formed two different clusters (Fig. 4) indicating the possibility of using genetic barcode to track the parasite to its origin. This view was further strengthened through testing of 12 parasite isolates with ‘unknown origin’ that was found to cluster with the other Sri Lankan samples, confirming that they are likely to be indigenous malaria parasites. Nine of the parasite isolates from Sri Lanka however, clustered with the South American cluster while 2 of the South American isolates were found to cluster with the Sri Lankan group (Fig. 4). Of the 9 isolates that clustered with the South American group, 8 were from the year 2010 (5 from Eastern Province, 2 from Southern Province, 1 from Northern Provinces), and one from year 2007 collected from the Eastern Province outbreak.

However, when only the Sri Lankan isolates were used in PCA, it did not show any clustering according to the year or the location from which the local parasite isolates were collected, potentially due to the limited sample size.

### Minor allele frequency (MAF)

Forty SNPs were studied in each sample sub-set, with the number of polymorphic SNPs varying each year (10, 13 and 7 polymorphic loci, respectively, in 2005, 2007 and 2010; mean = 10.00 ± 2.49). When the whole population was considered, 32 out of 40 SNPs (80%) had minor allele frequencies (MAFs) of > 0.1. When each sample sub-set was considered, the range of minor allele frequencies of the SNPs differed between the sample sub-sets of different years (from 0.0 to 0.5). Basic properties of the genetic nature of the parasite populations are summarized in Table 1.

**Table 1:** Genetic characteristics of parasite isolates collected between 2005–2011

	2005	2007	2010
No of isolates	8	23	9
No of SNPs	40	40	40
Proportion of multi-clone infections	25%	69.56%	22.22%
Mean number of pair-wise differences (in nucleotides ±SD)	4.821 (± 2.631)	4.771 (± 2.416)	1.400 (± 0.934)
Mean COI	1.142	1.25	1.136

### Complexity of infection (COI)

Nearly 70% (69.56%) of the sample sub-set from 2007 had at least two genetically distinct parasite strains (Table 1), and the proportion of multi-clone infections in the sample sub-set from 2007 was significantly higher (χ^2^ = 10.227, p = 0.017, adjusted residual ± 3.1) compared to that in the other years (25.0% in 2005, and 22.22% in 2010). The overall percentage of multi-clone infections for the entire sample set genotyped was 39.21%. Complexity of Infection (COI) represents the number of genetically distinct parasite strains simultaneously infecting a patient. The mean COI was 1.142, 1.25 and 1.136 for each sample sub-set from 2005, 2007 and 2010, respectively. Furthermore, out of 40 loci 37 (92.5%) were polymorphic when polyclonal samples were considered. This was comparable with the percentage of polymorphic loci in monoclonal samples (95%).

### Linkage disequilibrium (LD)

Significantly high (p < 0.05) LD was observed between several polymorphic loci of the sample sub-sets collected from 2005 and 2007. The maximum numbers of linked loci per polymorphic loci were 6 and 3 in these years, respectively. None of the polymorphic loci was in linkage to the sample sub-set from 2010.

### Genetic distances of the sample sub-sets from different years

**Fig. 5:**
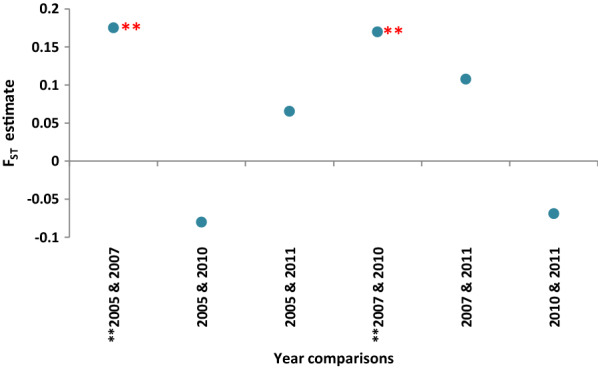
Pair-wise F_ST_ values obtained for the parasite sub-populations for each year. The parasite populations from 2007 showed a significant difference in their genetic make-up when compared with the populations from years 2005 and 2010

The pair-wise F_ST_ values estimated for all SNPs indicated that the sample sub-set from 2007 that originated from an outbreak in Trincomalee district differed significantly (p < 0.05) from the sample sub-sets from 2005 and 2010 (Fig. 5).

### Genetic structure of sample sub-sets

**Fig. 6:**
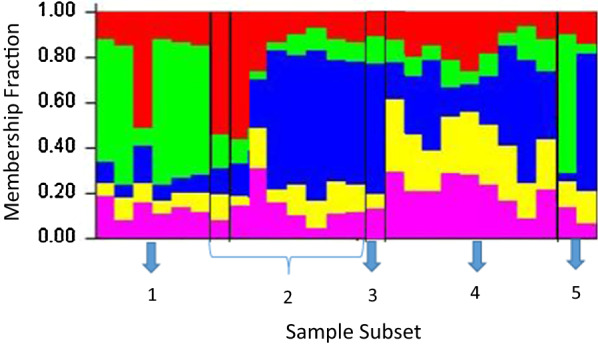
The population structure (the membership fraction for each sample sub-set) of *Plasmodium vivax* plotted according to the year in which the isolates were collected inferred from genotyping of parasite isolates (at *K* = 5). Each bar represents an isolate. Cluster 1-isolates from 2005 (n = 6), cluster 2-isolates from 2006 (n = 1) and 2007 (n = 7), cluster 3-isolates from 2009 (n = 1), cluster 4-isolates from 2010 (n = 8) and cluster 5-isolates from 2011 (n = 2). Seven isolates for which the year of collection is unknown and isolates with multi-clone infections (n = 19) were excluded from structure analysis

The structure analysis, which indicates the proportion of ancestry in each isolate, also revealed that sample sub-sets from the short epidemics 2007 (cluster 2) and 2009/10 (clusters 3 and 4) were more genetically different and diverse when compared to the sample sub-set from the early years, i.e., 2005 (Fig. 6).

## Discussion

The parasite isolates for this study were collected from 2005–2011 mainly from three provinces in Sri Lanka during the period of declining vivax malaria transmission. The genetic composition of a parasite population over time is known to reflect the changes in transmission intensity especially in *P. falciparum*. However, the genetic information derived of parasite populations could be of limited use in assessing population dynamics and transmission intensity in high transmission areas due to frequent multi-clonal infections (super infections) compared to low transmission areas [29]. On the other hand, the quality of the genetic data depends on the method of genotyping, i.e., although SNP barcoding method could be affected by the presence of high multi-clonal parasite populations, some methods, such as amplicon deep sequencing are not affected [30].

In the present study, the local *P. vivax* parasite population showed an overall high level of genetic diversity based on the 40-SNP barcode, COI and structure analysis, which was viewed in the context of low malaria transmission that prevailed during the study period. This confirms the observations made previously in Sri Lankan *P. vivax* populations using microsatellite markers [31]. Similarly in other geographical regions, it has been shown (using SNP markers) that *P. vivax* tends to maintain a higher genetic diversity even in areas with low transmission. Cui et al*.* suggested that observation could be due to a number of factors, including intrinsic biological properties of the vivax malaria parasite, such as relapses and early gametocytaemia which favour the cross-fertilization and meiotic recombination of distinct parasite genotypes within the vector. Furthermore, though rare, this genetic diversity could also be influenced by imported parasite strains carried by infected immigrants, as this can increase the genetic variability of the parasites through recombination in the mosquitoes [32]. The parasite isolates in the present study were collected during a period in which intense control programmes, especially residual spraying of insecticides for vector control, were in place throughout endemic areas of Sri Lanka. Consequently, the behaviour and distribution of vector mosquitoes may have been affected, which may have influenced the length of the extrinsic parasite life cycle, ultimately influencing the genetic diversity of the parasite population [33]. However, the connection between genetic diversity and the transmission intensity of malaria could be complex, with these two components having a non-linear relationship [34].

High LD is always prominent in areas with lower transmission intensities where genetic diversity is low, as the linkage of markers across chromosomes usually happens when the rate of sexual recombination is low. Sample sub-sets from 2005 and 2007 in this study demonstrated such a pattern. The co-occurrence of high LD with multi-clones under decreased transmission intensity was however unusual, although described previously as well by Karunaweera et al*.* and Gunawardena et al*.* using 14 polymorphic microsatellite markers [31, 35]. The results of the present study further confirm these previous observations. This scenario has also been observed in parasite populations in Brazil [36]. Therefore genetic linkage, especially in low transmission settings, can be a valuable tool to track parasites in populations both temporally and spatially [29].

One of the main findings in this study is that the local parasite population had a high proportion of multi-clone infections. Multi-clone infections arise when an individual is simultaneously infected by more than one parasite genotype. This can be due to co-infection of different parasite strains, viz*.* super infection, which normally occurs with high endemicity or as speculated by Cui et al. through somatic mutations during the course of infection [32]. Nearly 70% of parasite isolates in the sample sub-set from 2007 were multi-clone infections despite the reduced malaria endemicity. However, when the entire *P. vivax* parasite sample set was considered (n = 51), the overall percentage of multi-clone infections was 39.21%. This is a relatively lower value compared to those in other geographical areas with relatively higher vivax malaria transmission rates than in Sri Lanka, that include Vietnam (71.4%) [37], Colombia (60–80%) [38], and the Amazon Basin of Brazil (50%) [39]. However, the percentage multi-clone infections obtained for sample sub-sets from individual years (apart from the sample sub-set from 2007) and for the total sample set used were comparable to those observed in places with similar transmission dynamics (*P. vivax*), i.e., in low endemicity areas in South Asia, with 25.8% in some parts of Malaysia [40]; in post-intervention sites including Solomon Islands (27%) [41], Peruvian Amazon (34.7%) [42], and also in low-endemicity areas in South America with *P. falciparum* infections [43, 44]. In contrast, previous studies in Sri Lanka using microsatellite markers by Karunaweera et al. in 2007/2008 and Gunawardena et al*.* in 2014 reported higher percentages (60, 52, and 68%, respectively) of multi-clone infections [32, 34, 45]. Those samples however had been collected during 2003–2007, when malaria transmission was still relatively higher than the period during which the samples were collected for this study. The samples included from year 2010 had 22% multi-clone infections, during which time the transmission intensity was very low. These lower values may have contributed to the overall lower percentage of multi-clone infections in this parasite population. Moreover, relapses due to hypnozoites also could account for multi-clone infections in vivax malaria. However, the relapse rates of vivax malaria in these study sites are not well studied to infer the impact of relapses on multi-clone infections. Furthermore, a 2-week course of primaquine was widely used as a radical cure for vivax malaria in Sri Lanka, especially during the malaria pre-elimination phase; eliminating hypnozoites and thus preventing relapses, may have accounted for the low number of multi-clone infections during later years.

The use of several molecular markers increases the chance of detection of multi-clone infections compared to using a few or a single marker [31, 46].

The 40-SNP barcode (Fig. 3 and Additional file 3) revealed the presence of common alleles (haplotypes/barcode types) in parasite clusters from different years and in clusters from different areas, indicating a conserved and/or global distribution of certain parasite genotypes. Each isolate studied possessed a unique haplotype, but shared/common sub-sets of the barcode could be observed between the isolates within the same year as well as between the years. Having the shared/common sub-set of the barcodes in many isolates could be due to either clonal propagation or epidemic expansion (or both). Although the barcode SNPs on their own are not subjected to selection, the parasite lineages might be associated with other phenotypes that may be under selection pressure, e.g., reproductive success (gametocyte formation, multiplying or recombining inside the vector, etc.). Furthermore, the immune factors in humans (e.g., lack of immunity against a genotype that causes rapid expansion without any association with anti-malarial usage) or other stochastic events, could also affect the allele frequencies of those SNPs [14]. The geographical and temporal distributions of selected parasite genotypes might also be influenced by the seasonal/temporal population dynamics of host anopheline mosquito strains, as reported by previous studies describing differences in the susceptibilities of vector mosquitoes to different genetic strains of the parasite [47].

Patient details available did not specify any travel outside of the country for any patient from whom these samples were obtained and, therefore, the isolates were assumed to be indigenous. Out of the 39 isolates with records of the location from which they were collected, 30 isolates were included in a single cluster in PCA, suggesting having a similar genetic structure. There were 12 isolates of which the location of the patient was unknown and in the PCA these isolates clustered with the other Sri Lankan samples, supporting the assumption that those also could be indigenous isolates. Interestingly, 9 isolates (out of which 8 were from year 2010) clustered together with the South American group. It could be speculated that these isolates were imported cases. However, they have been reported from 3 provinces (although the majority were from the Southern province, n = 5). Since no travel histories were indeed available, such speculations remain unconfirmed.

Although the 40-SNP barcode revealed clustering of genetically similar parasites when compared with isolates from other global geographic regions by PCA, it could not distinguish prominent parasite clusters geographically or temporally within the Sri Lankan isolates, indicating the genetic similarities of the parasite isolates used in the analysis. Interestingly, the sample sub-set from 2007, which was collected during a short-term malaria outbreak in Trincomalee, was more genetically diverse compared to the parasite populations from other years. Sri Lanka is a small island, with relatively low physical and socio-demographic barriers, which enables mixing of parasites with different genetic profiles; thus, there is limited potential to preserve geographically isolated parasite populations with distinguishable genetic features. However, this may account for the high genetic diversity in Sri Lankan *P. vivax* populations. Therefore, the 40-SNP barcode may not be sensitive enough to capture the finer scale diversity among highly clonal parasite isolates, as reported in this study. Further studies warrant the development of an extended barcode comprising a greater number of SNPs to differentiate regional parasites with high rate of multi-clone infections.

The origin of a focal outbreak is likely to be a common source and therefore, one might expect to find genetically similar parasites in such a setting. However, the study findings indicated a higher level of genetically diverse groups of isolates within the outbreak when compared to the sample sub-sets from other years. The decreased diversity of sample sub-sets from 2005, 2010, and 2011 may have been influenced by the low transmission/endemicity, with fewer opportunities for sexual recombination between genetically distinct parasites circulating during that period, while the parasite population from the short epidemic (in 2007) may have had a higher chance of recombination with genetically diverse parasites during the short epidemic that occurred in the Trincomalee district, resulting in the high level of genetic diversity observed in this sample sub-set from 2007. Gunawardena et al*.* in 2010 used microsatellite markers to study the genetic diversity of the vivax isolates from Sri Lanka, reporting that isolates collected during the 2007 malaria outbreak in Trincomalee district demonstrated distinct differences in their genetic make-up compared to that in parasite isolates collected during other years [31]. The results of the current study confirm this observation.

One of the major drawback of this study is the limited numbers contained within the 2006 and 2011 sub-sets, i.e., one sample and two samples, respectively, in 2006 and 2011. However, those sample sub-sets were largely excluded from the analyses, but used only in selected analyses (viz. construction of the genetic barcode) where the outcome is independent of the sample number contained within the sub-set. Furthermore, the information derived from haplotype analyses and F_ST_ estimates in this study might not be very informative owing to the small sample size and the high variation of allele frequencies observed between the sample sub-sets from different years. Therefore, future studies with a large number of samples will warrant more accurate and robust results.

More importantly, this study demonstrates the potential significance of using genetic barcodes to aid in distinguishing parasite populations, enabling tracking of imported malaria cases that pose a threat during the post-malaria elimination phase in Sri Lanka and elsewhere.

## Conclusions

This study provides supportive evidence for the potential utility of a genetic barcode that could aid in confirming indigenous malaria cases. In-silico analysis revealed that isolates with unknown origin clustered with Sri Lankan isolates in PCA, supporting the likelihood of these samples being indigenous. This also provides the ability to identify imported malaria cases that pose a threat during the post-malaria elimination (prevention of re-introduction) phase in Sri Lanka. The high percentages of multi-clone infections in the sample sub-sets and the mean COI values/analysis with STRUCTURE (V 2.3.4) software suggests an overall high level of genetic diversity within an environment of low malaria transmission that prevailed during the study period. This effect was more pronounced during the short malaria epidemic in 2007. However, studying the parasite variations within the country needs a more sensitive barcode, probably with a higher number of SNPs to distinguish between the indigenous populations. However, the 40-SNP *P. vivax* barcode was able to identify common/shared sub-sets of barcodes in parasite sample sub-sets from different years and within sample sub-sets from different areas.

## Supplementary information

**Additional file 1.** Details of the parasite isolates used in the study; ID number, year and location of collection, and clonality.

**Additional file 2.** Details of the genotyped SNPs; SNP ID, chromosome position, gene ID and the genetic properties/effect.

**Additional file 3.** SNP genetic barcode; Genotyped SNPs arrange according to the place of collection.

## Data Availability

The datasets used and/or analysed during the current study are available from the corresponding author on reasonable request.

## Abbreviations

WHO: World Health Organization
HRM: High-resolution melting
SNP: Single nucleotide polymorphism
PCR: Polymerase chain reaction
WGA: Whole genome amplification
T_m_: Melting temperature
MAF: Minor allele frequency
LD: Linkage disequilibrium
F_ST_: Fixation Index
COI: Complexity of infection
