# Supplementary material for: Use of a Plasmodium vivax genetic barcode for genomic surveillance and parasite tracking in Sri Lanka
Source: Malar J. 2020 Sep 21;19:342. doi: 10.1186/s12936-020-03386-3 (PMC7504840; doi:10.1186/s12936-020-03386-3)
Supplement: Supplementary file 3 — Additional file 3. SNP genetic barcode; Genotyped SNPs arrange according to the place of collection. [file 12936_2020_3386_MOESM3_ESM.pdf]

Province	Year	Code	1	2	3	4	5	6	7	8	9	10	11	12	13	14	15	16	17	18	19	20	21	22	23	24	25	26	27	28	29	30	31	32	33	34	35	36	37	38	39	40
Reference Allele			C	C	A	A	C	A	T	G	C	C	C	T	A	T	C	C	T	G	C	C	T	T	C	C	G	G	T	G	C	C	G	C	G	T	C	G	C	T	A	C
99	99	1	C	C	A	G	T	G	T	G	T	C	C	C	A	C	T	T	T	T	C	T	C	C	T	C	A	A	C	G	T	T	A	T	G	T	C	G	T	T	G	C
99	99	2	C	C	A	A	T	G	T	G	T	C	C	C	A	C	T	X	T	T	C	T	C	X	T	C	A	A	T	G	T	T	A	X	G	C	C	G	T	T	G	C
99	99	3	C	C	A	G	T	G	C	G	T	T	C	C	A	C	T	C	G	G	C	T	C	C	T	C	A	A	T	G	T	C	G	T	G	C	C	G	T	T	A	C
99	99	248	C	C	A	A	T	G	T	G	T	C	C	C	A	C	T	T	T	T	C	T	C	C	T	C	A	A	T	G	T	T	A	T	G	T	C	G	T	T	G	C
99	99	520	C	C	A	A	T	G	T	G	T	C	C	C	A	C	T	T	T	T	C	T	C	C	T	C	A	A	T	G	T	T	A	T	G	T	C	G	T	T	G	C
99	99	4793	C	C	A	A	X	G	T	G	T	C	C	C	A	C	T	T	T	T	C	T	X	C	T	C	A	A	T	G	T	T	A	T	G	T	C	G	X	T	G	C
99	2005	12	C	C	A	A	T	G	T	G	T	C	C	C	A	C	T	C	T	T	C	T	C	C	T	C	A	A	T	G	T	T	A	T	A	T	C	G	T	T	G	C
99	2005	15	C	C	A	G	T	G	T	G	X	C	C	C	A	C	T	T	T	T	C	T	C	C	T	C	A	A	T	G	T	T	A	T	G	T	C	G	T	T	G	C
99	2007	23	C	C	A	A	T	G	T	G	T	C	C	C	A	C	T	X	T	T	C	T	C	C	T	C	A	A	T	G	T	T	A	X	G	T	C	G	T	T	G	C
99	2007	24	C	C	A	A	T	G	T	G	T	C	C	C	A	C	T	T	T	T	C	T	C	C	T	C	A	A	T	G	T	T	A	T	G	T	C	G	T	T	G	C
99	2011	MR4783	C	C	A	A	T	G	T	G	T	C	C	C	A	C	T	T	T	T	C	T	C	C	T	C	A	A	T	G	T	T	A	T	G	T	C	G	T	T	G	C
99	2011	MR4785	C	C	G	G	C	A	C	A	T	C	T	T	A	T	C	C	G	G	X	C	C	C	C	C	A	A	T	G	T	T	A	T	A	C	C	G	T	T	G	C
Eastern	2006	22	C	C	A	A	T	G	T	G	T	C	C	C	A	C	T	T	T	T	C	T	C	C	T	C	A	A	T	G	T	T	A	T	G	T	C	G	T	T	G	C
Eastern	2007	25	C	C	A	G	T	G	T	G	T	C	C	C	A	C	T	T	T	T	C	T	C	C	T	C	A	A	T	G	T	T	A	T	A	T	C	G	T	T	G	C
Eastern	2007	34	N	C	A	G	T	A	C	G	C	T	C	C	A	C	X	C	G	G	C	T	C	X	C	A	N	A	T	G	T	C	G	T	A	C	C	G	T	T	N	C
Eastern	2007	G115	C	C	A	G	T	A	X	G	C	T	C	X	A	C	T	C	G	G	T	T	C	C	C	A	A	A	T	G	T	C	G	T	G	T	X	G	T	T	A	C
Eastern	2007	T1	C	C	A	A	T	A	T	G	T	C	C	C	A	C	X	X	T	T	T	T	C	C	T	C	A	A	T	G	T	T	X	X	G	T	C	G	T	T	G	C
Eastern	2007	S2 pilot screen	N	C	A	G	T	G	N	A	T	N	C	T	A	T	T	N	G	G	N	T	T	C	T	C	G	G	T	G	N	N	A	C	A	T	C	G	T	C	G	N
Eastern	2007	S5 pilot screen	C	T	N	G	T	A	T	A	T	C	N	N	G	T	T	N	N	N	C	C	T	C	N	N	N	N	T	G	N	N	N	N	G	N	C	G	T	N	N	C
Eastern	2007	S6 pilot screen	C	T	G	G	N	A	C	A	T	C	C	C	G	T	T	T	T	G	C	C	T	C	C	C	A	A	T	G	T	T	G	C	A	N	C	G	T	T	G	C
Eastern	2007	S7 pilot screen	C	T	A	G	C	G	C	A	C	T	C	T	A	C	T	T	T	G	T	T	T	T	C	C	A	G	C	A	C	C	G	C	G	C	C	G	T	C	G	C
Eastern	2007	S8 pilot screen	C	T	G	G	T	G	C	A	T	T	C	C	A	T	T	C	T	G	C	C	T	C	C	C	A	A	T	G	N	T	G	C	A	C	C	G	T	T	G	C
Eastern	2007	S9 pilot screen	C	N	N	N	N	N	N	A	T	N	N	N	N	N	T	N	T	T	C	N	T	N	N	N	A	N	T	N	N	T	N	C	N	N	C	G	T	T	N	C
Eastern	2007	S10 pilot screen	C	C	A	A	C	A	C	A	C	T	T	N	G	C	T	T	G	G	C	T	T	T	T	C	A	G	C	A	N	T	A	C	G	N	C	G	T	T	G	C
Eastern	2007	S12 pilot screen	N	C	G	G	T	G	C	A	T	T	C	C	N	N	C	C	G	G	T	T	T	C	C	C	A	A	T	G	N	T	G	T	G	N	N	G	N	T	N	C
Eastern	2007	S13 pilot screen	C	C	G	G	T	A	C	A	T	C	C	C	A	T	T	C	G	G	N	T	T	C	C	C	A	A	T	G	T	T	G	T	G	C	C	G	N	C	A	C
Eastern	2007	S14 pilot screen	C	C	A	A	T	A	C	G	T	N	C	T	G	C	T	N	T	G	T	C	C	N	T	C	A	G	T	G	T	T	G	T	G	N	C	G	T	T	A	C
Eastern	2007	S16 pilot screen	C	C	G	A	T	G	C	G	C	T	C	T	G	C	T	T	T	G	T	C	C	T	T	C	A	G	C	G	T	T	G	T	G	C	C	G	T	T	A	A
Eastern	2007	S17 pilot screen	C	C	G	G	T	A	T	G	C	N	T	C	A	C	T	C	G	G	T	C	C	C	C	C	A	G	C	G	T	C	G	C	A	C	C	G	T	T	G	C
Eastern	2010	90	C	C	A	G	T	G	C	G	C	T	C	C	A	C	T	C	G	G	T	T	C	T	T	C	A	A	T	G	T	C	G	T	G	C	A	G	T	T	A	C
Eastern	2010	T2	C	C	A	A	T	G	T	G	T	C	C	C	A	C	T	T	T	T	T	T	C	C	T	C	A	A	T	G	T	T	A	T	G	T	C	G	T	T	G	C
Eastern	2010	T4	C	C	A	A	T	G	T	G	T	C	C	C	A	T	T	C	T	T	T	T	C	C	T	C	A	A	T	G	T	T	A	T	A	T	C	G	T	T	G	C
Eastern	2010	T7	N	C	A	N	T	G	N	G	N	N	C	C	A	C	T	N	N	T	C	T	C	C	T	C	A	A	T	G	T	N	N	T	G	N	C	G	T	T	G	C
Eastern	2010	S3 pilot screen	C	T	A	G	C	G	C	A	T	C	C	T	G	T	T	T	G	T	T	C	T	C	C	C	A	G	T	G	C	T	G	C	A	T	C	G	T	T	A	C
North Western	2005	5	C	C	A	G	T	G	C	G	T	T	C	C	A	C	T	C	G	G	T	T	C	T	T	C	A	G	T	G	T	C	G	T	G	C	C	G	T	T	A	C
North Western	2005	6	C	C	A	G	T	G	C	G	C	T	C	C	A	C	T	C	G	G	T	T	C	T	T	C	A	A	T	G	T	C	G	T	G	C	C	G	T	T	A	C
North Western	2005	7	C	C	A	G	T	G	C	G	C	T	C	C	A	C	T	C	G	G	X	T	C	T	T	C	A	A	T	G	T	C	G	T	G	C	C	G	T	T	A	C
North Western	2005	8	C	C	X	A	T	G	T	G	T	C	T	C	X	C	T	T	T	T	C	T	C	X	T	C	A	A	T	X	T	T	A	T	G	T	C	G	T	T	G	C
North Western	2005	9	C	C	A	A	T	G	T	N	T	C	C	C	A	N	T	T	T	T	C	N	C	C	T	C	A	A	T	G	T	T	A	T	A	T	C	G	T	T	G	X
North Western	2005	16	C	C	N	A	T	N	T	G	T	C	C	N	A	N	T	N	N	N	C	N	C	C	T	C	A	A	T	G	T	T	A	T	A	T	C	G	T	X	G	C
Southern	99	G15	C	C	A	N	X	A	C	G	T	T	C	C	A	T	T	C	N	G	X	T	C	C	C	A	A	A	T	G	T	C	X	T	N	T	C	G	T	T	A	X
Southern	2009	G23	C	C	A	G	T	G	C	G	C	T	C	C	A	T	T	C	G	G	T	T	C	T	T	C	A	A	T	G	T	C	G	T	G	T	C	G	T	T	A	C
Southern	2009	S1 pilot screen	C	T	G	G	T	A	C	G	N	T	C	T	A	T	T	T	G	G	T	T	C	C	T	C	A	A	T	G	T	T	A	T	G	N	C	G	T	T	G	A
Southern	2009	S11 pilot screen	T	C	A	G	C	A	C	N	C	C	C	C	A	N	T	N	G	G	N	T	T	C	C	C	A	A	T	G	N	T	G	T	G	C	C	G	C	C	A	C
Southern	2010	95	C	C	A	A	C	A	T	G	T	T	C	C	A	C	T	C	G	G	C	T	C	T	C	C	A	A	T	G	T	T	G	T	A	C	C	G	C	T	G	C
Southern	2010	MR4775	C	T	G	N	N	N	N	A	C	N	T	T	A	N	C	C	T	N	C	C	C	X	N	N	N	N	T	N	T	T	A	N	A	T	X	G	T	T	N	C
Southern	2010	620	C	C	A	A	T	G	T	G	T	C	C	C	A	C	T	T	T	T	C	T	C	C	T	C	A	A	T	G	T	T	A	T	G	T	C	G	T	T	G	C
Southern	2010	S18 pilot screen	C	C	A	G	T	A	T	G	C	T	C	T	A	C	T	C	G	G	C	T	T	T	T	C	G	G	T	G	C	T	A	C	A	C	C	G	T	C	G	C
Southern	2010	S19 pilot screen	N	T	G	A	T	A	N	N	N	T	T	N	A	C	T	C	T	G	T	T	T	T	C	C	A	G	T	G	N	T	A	T	G	C	N	G	T	C	G	A
Uva	2009	S15 pilot screen	C	C	N	A	T	A	C	G	C	N	C	T	G	C	T	N	T	T	T	C	T	T	T	C	A	G	T	G	T	C	G	T	G	C	C	G	T	T	A	A
Northern	2010	S4 pilot screen	C	T	N	G	T	A	C	G	T	C	C	C	N	T	T	T	G	G	T	C	T	C	T	A	G	G	T	G	T	C	G	T	G	N	C	G	T	T	A	C
